# Lived Time Disturbances of Drug Addiction Therapy Newcomers. A Qualitative, Field Phenomenology Case Study at Monar-Markot Center in Poland

**DOI:** 10.1007/s11469-016-9680-4

**Published:** 2016-06-21

**Authors:** Marcin Moskalewicz

**Affiliations:** 1 Department of the History of Medical Sciences, Poznan University of Medical Sciences, Poznan, Poland; 2 Collegium Helveticum, ETH Zurich and University of Zurich, Zurich, Switzerland; 3 Res Publica Foundation, Warsaw, Poland

**Keywords:** Temporality, Phenomenology, Temporal experience, Traumatic past, Time horizons, Dissociated future, Qualitative

## Abstract

The aim of this study was to qualitatively assess the disturbances of lived time in individuals with multiple drug dependencies. The research was conducted at Monar-Markot Center for Humanitarian Aid in Roznowice (Poland) in 2014 through direct, non-disguised observational study in a therapeutic community setting. Overall, 10 clients with multiple drug dependencies forming a newcomers group participated in. They previously abstained from usage for negligible periods of time. The measurements included participant observation of collective time regimes at the center; group discussions; written accounts by clients; Zimbardo Time Perspective Inventory; semi-structured interviews. It was found that the clients experienced difficulties in following a strict therapeutic temporal regime, and they also showed a concomitant need to accelerate time as it passed. They also suffered an unpleasant domination by traumatic past within lived time. Their time horizons appeared significantly shortened and their planning capacity impaired, while a distant (dissociated) future was fantasized about in a realistic manner. Altogether, their disturbances of lived time consisted of the propensity to overemphasize their past dimensions at the expense of their future, while a gap between a close and a distant future appeared.

## Lived Time and Phenomenology

This study seeks to identify the unusual lived time experiences of multiple drug-addicted individuals, while in a sober state. This study is not concerned with the examination of the distorting effects of drugs on the experience of time, instead it will focus on the temporal structure of consciousness analyzed vis-à-vis the intentional world (i.e. not some objective, external reality, but the world as present within the consciousness (Gallagher 2012)). From the perspective of phenomenology, time is not simply a datum of consciousness, but its necessary underlying phenomenon – a lived condition of possibility of objects and of events (Heidegger 1962; Husserl 1991; Lohmar and Yamaguchi 2010; Merleau-Ponty 1962). Lived time consists of three dimensions, past, present and future, overlapping within what William James called a “specious present” (James 1992). Their mutually experienced relationship is constituted through the reflexive activities of a subject (Minkowski 1970). The focus of this research study is this primary, temporal structure of experience, as observed and experienced from within.

## Temporal Experience in Addiction

Lived time as a notion has been used in theoretical and empirical research in both normal and pathological experiences for almost a century (Spiegelberg 1972). Regarding addiction, it has been argued that people addicted to alcohol live in a hopeless, present time (Gliedman 1956). They may experience a loss of continuity in their lives, as their time breaks into dissociated moments (von Gebsattel 1954). In contrast to objectified addiction, lived addiction was interpreted as a problematic form of existence that focuses on the present (Copoeru 2014). Regardless of and in addition to the ontical, neurophysiological crisis, an addicted existence – also in a state of physiological sobriety – is a distorted way of being in the world, for both the lived body and the lived mind (Trujillo 2004). It was claimed, that addicted existence entails limited temporal horizon and is primarily directed toward the now (Jarvinen and Ravn 2015; Kemp 2009). The addicted person is overwhelmed by the present and by short-term desires (Ruckenstein 2013). In extreme heroin cases an addicted person may experience this present without any future (Reith 1999). Phenomenologically speaking, it is not a genuine present, because it lacks a properly extended “here and now” aspect. It is merely an empty, momentary now, without the dimensions of past and future (Di Petta 2014).

It has been observed that people addicted to heroin in particular have shortened time horizons; this may explain their persistent use of drugs, despite the long-term negative consequences attributed to its use (Petry et al. 1998). Active opiate users also have a truncated future perspective when compared with non-active users (Alvos et al. 1993). Correlations between time perspective and substance use, based on the Zimbardo Time Perspective Inventory (ZTPI), have been reported. The most predictive factor for substance use was a present hedonistic attitude, while the focus on the future indicated an aversion to risky behaviors (Keough et al. 1999). Furthermore, a negative link has been found between a high future time perspective and cannabis use (Apostolidis et al. 2006). People addicted to alcohol and drugs had a lower degree of orientation to the hedonistic future and present, and a higher degree of orientation to a negative past than the general population (Klingemann 2001). In a different cultural context, alcohol addicted patients at the beginning of the therapy session preferred a past and a present temporal orientation rather than a future one, while the latter was found to be beneficial for finishing the therapy by men (Chodkiewicz and Nowakowska 2011). Some marginal significance was also observed for a future temporal orientation being a predictor of long-term alcohol abstinence (Lennings 1996). Recent studies have confirmed that the more people are focused on a present hedonistic perspective, the more they report substance use, while the more they are focused on a future perspective the less they report substance use (Fieulaine and Martinez 2010). A future orientation of adolescents on ZTPI was indirectly, inversely related and a present orientation positively related to early-onset addictive substances use (Wills et al. 2001). Alcohol abusers also had a less extensive and a less coherent future perspective than social drinkers (Smart 1968).

At the extreme, unable to imagine the future and not being able to experience a smooth, linear passage of time, the addicted person may experience a constant and compulsive repetition of the same. A loss of the past as well as a number of personal losses plus a limited future horizon may push him or her into regressive cycles, in which losses become worse and worse, and future horizons become more and more limited (Erdos et al. 2009). It has been argued phenomenologically that prior to their recovery the addicted persons’ personality is unable to develop in objective time and is thus reduced to the level of cyclical temporality (Zutt 1963a). The number of possibilities of the future becomes radically reduced to just one – that of being high and free of craving (Trujillo 2004). Ultimately, such a constant repetition may be experienced as a customary and natural self-sustaining bodily function (Schlimme 2010).

When it comes to the social dimension of temporality, addiction is defined as a disorder in the daily rhythms of mainstream society, and a failure in balancing the short and long-term shared time cycles (Ruckenstein 2013). For example, being out of synchrony with social time structures, young cannabis users suffered concomitant marginalization (Jarvinen and Ravn 2015). In a word, de-synchronicity is not healthy, it entails being unwell (Kemp 2009).

## Ideal-Typical Model of Lived Time in Addiction

On the basis of existing research, the following ideal-typical picture of the lived time of people suffering from addiction can be proposed: its present dimension is not extended and meaningful, but merely momentary and elusive (it lacks a past and a future dimension), in the long run it tends to become cyclical and regressive, leading to the repetitive existence of dissociated moments, while desynchronization with mainstream, collective temporality takes place.

This ideal-typical model is obviously abstract and does not concern existence in every given case of addiction. Nevertheless, it may explain why people with addictions tend to value immediate benefits above delayed rewards and why they continue using drugs despite the long-term negative consequences associated with their use. The quality of the lived time in addiction changes during recovery, which was temporarily described as opening future possibilities (Banonis 1989). The treatment of addiction has been found to significantly alter the time perspective both through quantitative (ZTPI) and qualitative analyses (Davies and Filippopoulos 2015). In the post-treatment stage clients had their negative past perspective and present hedonistic perspective lowered, and their future perspective increased. They also had a greater sense of being present, were less disorganized and had better control over the future.

## Methods

### Setting

The following study aims to complement the picture presented above through a focused phenomenological lived time analysis. The study was conducted in 2014 at Monar-Markot Center for Humanitarian Aid, a residential addiction rehabilitation center based on therapeutic community ideals, located in Roznowice, Poland. While at the center the clients contact time with the outside world was limited. Since the therapeutic community is supposed to replace society, the setting was assumed to be representative of the real life-world in the phenomenological sense, and not just an artificial environment. Time in closed environments, especially when it becomes structured or even oppressive, can be explicitly and consciously experienced, thus giving us access to what was previously only pre-reflectively apprehended (Cope 2003). Without this assumption, the results can be interpreted as pertaining merely to the study group’s adaptation to the new circumstances at the residential center, and not to the structure of their lived time in general.

### Participants

There were forty or soclients in the long-term (10–12 months) therapy program. The focus was on the “novitiate” group, which consisted of ten clients resident at the center for up to 2 months. The novitiate is the first stage for freshmen at therapy, which follows the “observation” stage before therapy (a probationary week before deciding whether or not to join the therapeutic community). Novitiates are full members of the community. They meet the conditions set for full drug and sexual abstinence and must follow the strict rules set by the community regarding their behavior.

The novitiate group (male:female = 7:3; median age = 27.5; all Polish and Caucasian; abstinence minimum two weeks and maximum 2 months) was chosen based on the assumption that the clients, even if physiologically sober retained their old behaviors and ways of thinking. All the members of this group were diagnosed with multiple drug use and drug dependence (ICD-10: F19.2). For three members of the group the predominant drug was alcohol, while for the others it was a mixture of alcohol, cannabinoids, stimulants, hallucinogens as well as other designer drugs, with unknown combinations also possible because the ability to determine the mixture was reliant on self-reporting. Only one member of the group was not addicted to alcohol, and only one was a former opiate user with 15 years of abstinence from heroin. Eight clients were in therapy for the first time while two had been previously treated. The initials of the novitiates have been changed to protect their identities.

### Methods

The following five research methods were used in an overlapping, chronological order.

- A) The researcher’s participant observation as a therapeutic community member (having the status of addiction therapy trainee during internship), which entailed taking part in daily, obligatory common meetings with the community. The observation was focused on superimposed forms of ordered time in the center.
- B) Discussions concerning the problem of the experience of time, conducted during two group therapy sessions with the novitiate group of approximately 2 h in length . The clients were asked about their experience of the strict time regime in the center. They were also invited to comment on their experience of time in general and on how they imagined their future in therapy.
- C) Written accounts on the subject: “How do I cope with functioning within the daily schedule in the center”? In order to detect any underlying and not necessarily explicit temporal experience issues, the question was deliberately asked without mentioning the subject of time.
- D) Zimbardo Time-Perspective Inventory (ZTPI), the most widely used psychometric measure of subjective time in its five, abstract dimensions (P. Zimbardo and Boyd 2008; P. G. Zimbardo and Boyd 1999). Even though this questionnaire could not give an account of phenomenologically understood lived time (Moskalewicz 2015), it was considered valuable as it aided the semi-structuring of the final interviews. Information regarding particular clients, especially if they deviated heavily from the population average in a given time orientation, was one of the main focal points in the interviews.
- E) Semi-structured individual interviews, explicitly concerning the subject matter of lived time with a focus on an individual’s temporal field (Finlay 2011). All interviews were collected in person by the researcher, in a room at the center kept for this purpose alone, each lasting for about an hour. In accordance with the phenomenological principle to bracket the existing knowledge about the world – in this case concerning what is generally known regarding time experience in addiction – with all that was pressuposed during the interviews was the knowledge gathered in stages A-D. Each interview was roughly divided into three parts: lived past, lived present and lived future. The questions concerned both explicit experience of each of the dimensions of time (e.g. “do you often think about the past?”; “how does your time passes?”; “how do you imagine your future?”) and implicit experience of time (i.e. time as underlying phenomenon of any conscious experience – questions such as: “How do you feel when X happens?”). Questions were slightly different for each individual, based on what was already known about that person’s experiences. The conversation was kept as natural as possible so that the experiences shared would not be about one’s abstract knowledge of one’s past, present and future, but about his or her lived time (using only explicit questions might result in answers pertaining merely to how one *understands the concepts* of past, present and future in one’s daily life, which might be different from how one actually, and possibly without being explicitly aware of it, *lives* his past, present and future). During the collection of data concerning lived future semi-quantification was used in order to assess the length of clients’ temporal horizon in a dialogue (on the basis of questions such as: “what are you doing tomorrow?”; “what are your plans for next week?”).

### Order of Research

Since time, even if appropriated individually, has a social dimension (Levine 1997), the research started with an observation of the collective, temporal life-world at the center (A). The next stage (B) involved knowing the novitiate group better, as well as gaining credibility and trust. During stages A and B handwritten notes were taken – they were coded and analyzed later, together with the clients’ written accounts (C), but before the interviews (E), in order to sketch a preliminary structure for the latter. The intention for including ZTPI (D) was to minimize the instance of bias by adding an objective measurement, even if only to serve as guidance for a particular interview. Altogether, stages A-D were treated as being preparatory for the interviews (E), which were considered the most relevant of the research methods, providing indirect yet the closest access to clients’ subjective experiences of time.

### Interpretation

Since lived time is by definition non-measurable and laden with pre-reflective meanings, it can ultimately be studied only through interpretative phenomenological analysis, while all objective measures give only a sense of a client’s time perception (i.e. an experience of time as an object). Such an analysis has been successfully applied in healthcare research (Lindseth and Norberg 2004; Shinebourne and Smith 2011) and given the specificity of the subject matter was chosen as the most adequate (Finlay 2011). Interpretative phenomenological analysis is concerned with how people make sense of their experiences, in their own terms, and what significance it carries within (Smith et al. 2009). During discussions and interviews, the researcher (being himself trained in phenomenological philosophy and having worked on the issue of the experience of time before), was focused on detecting essential, structural aspects of the individual temporal experience of clients. This involved abandoning any presuppositions, being highly emphatic, and paying attention to details not necessarily explicitly having to do with temporal experience. The linguistic materials were collected in Polish during the interviews, and categorized in advance to lived past, lived present and lived future. A lot of materials were disregarded as already irrelevant at this stage, as not pertaining to temporal experience at all. The only exception to the principle of non-measurability of lived time was the semi-quantification of the future horizon of clients. Subsequently, the research notes concerning the relevant materials were closely read and coded. After a transcription into a digital format, they were cross-analyzed by the researcher in order to detect common themes concerning each of the dimensions, while other incidental themes have been disregarded (Van Manen 1990). Taking advantage of the conceptual framework of phenomenology of lived time the researcher was concerned with: (1) detecting, if possible, essential aspects of each of the dimensions of time (such as the “dissociated future” and the “traumatic past” – see the results section), and (2) identifying, if possible, the subjective sense of the flow of time (acceleration versus slowing). In this way, the interpretation that was already taking place during the interviews (which were only semi-structured and were sometimes unpredictable) was supported by a more distanced analysis. At this stage, selected materials were translated into English. In addition to common themes, some idiographic insights have been intentionally preserved in order to give an account of individual life-world quality (see below). Most of the literature search was conducted after the actual study took place so that any overlap of themes between the study and state of research is due to the phenomena themselves and not the researcher’s unconscious imputations.

## Results

### Ad. A

Three temporalities organizing life in the center have been distinguished and named as such by the researcher: (1) circular, (2) linear and (3) presentist. The circular and linear temporalities stemmed explicitly from the treatment program employed at Monar-Markot, while the presentist has been added.

#### Ad. 1

A circular, 24-hour cycle contains a strict plan of the day. The time in the center is fully organized, with the exception of leisure breaks and free time on Sundays. Since clients are clearly unable to organize time by themselves, everyone is forced to follow a collective temporal regime set by the community. The strictly ordered daily rhythm includes; waking up, exercises, eating, working, individual and group therapy sessions, leisure breaks, cultural events and workshops, and ends with a curfew. Some cases of visible desynchronization of clients have been observed. An extreme example concerning the following of ordered clock time is the behavior of C (m/22). He forgets about meeting with the researcher, recalls this fact later but forgets the exact hour. After being reminded and rescheduled for half an hour later he does not come. When he meets the researcher searching for him in the corridor he exclaims, “I forgot!” During one of the therapeutic community meetings he „loses time” during a welcome ritual: when it is his turn to say “hello”, he forgets about it, even though a second before he was observing people doing this before him, and even then it seemed as if he had been preparing for his turn.

#### Ad. 2

Linear temporality of the whole therapy, divided into five stages: 1) observer (a week); 2) novice (2 months); 3) household member (4 months); 4) organizer (2 months); 5) monarian (3 months); each stage being associated with a number of specific tasks. Competences required at each stage are described on huge boards hanging in the common room where therapeutic community meetings take place daily. These boards constantly remind the clients about the content of each stage, their position in the group hierarchy, and the therapeutic progress. Each stage entails both new privileges and new responsibilities. Getting to the last stage of a monarian entails the privilege of not having to follow the day plan, but also the responsibility of organizing a day for oneself as well as writing a detailed and structured “recovery plan” for leaving the center, including a plan B. Even though these stages are relatively independent of the objective calendar, they constitute the most objective measure of time during the year. Each transition to the next stage is a small ritual: it involves coming to terms with one’s past in public, speaking about gains and leftovers as well as the planning of the future on paper. It has been observed that the clients longed for the further stages, which were a standard subject during small talk. Moreover, the hierarchy among them and their sense of self also had to do with the above mentioned stages. This fact was also represented spatially, as the observers and the novices were not allowed to sit on the chairs during therapeutic community sessions, thus visualizing the hidden hierarchy in space.

#### Ad. 3

Presentist temporality is embodied by a bell hanging in the corridor. Whenever something unusual or troublesome happens, every member of the community has the right to ring the bell in order to convene an extraordinary meeting. The ringing bell clearly disrupts whatever activity one is engaged in during the day, and forces an abrupt change of plans in order to focus on some important, actual problem. It was reported to be particularly annoying to some clients. B (f/28): “like cold water being poured over my head”. M (f/33): “I could not even finish the other half of my cigarette!”

### Ad. B

All participants were willing to share their experiences. Seven reported difficulties in keeping up with the daily schedule and showed signs of desynchronization. Five clients expressed the need to accelerate the passing time because it went by too slowly (see later section: *slow passing*). Clients also complained about the weekends and had problems with organizing their free time in general (even if there were about 2 h of leisure breaks during week days).

Half of the clients reported major difficulties in coping with free time, especially free time requiring self-organization K (m/31): “I do not know what to do with myself (…). I have problems organizing free time, I wander without a purpose”. L (f/36): “I cannot have too much free time (…). Sunday is a tragedy for me”. S (m/27): “I hate these Sundays (…). I always recall distaste after Saturday night parties (…). Sunday in front of me… an empty day”.

The future in therapy was seemingly imagined positively since they identified themselves with the therapeutic program and were longing for the privileges associated with the further stages. Nevertheless, when asked about concrete scenarios, the clients were unable to come up with anything precise. They were not planning anything, but waiting for a plan to be given to them by some external power. M (f/33): “Time is one huge mess (…). I am learning the order and planning”. F (m/25): “It is not about privileges but about someone telling me what I am supposed to do with myself”.

### Ad. C

All participants responded positively when asked to prepare written accounts. Ultimately, eight clients handed in their texts. The results of participant observation (A), discussions (B) and written accounts (C) regarding difficulties in keeping up with the schedule have been objectified together in Table 1 under the label “desychronization” according to a yes/no alternative. All eight clients reported difficulties in coping with the schedule. However, two of them showed mixed feelings, claiming to experience severe adjustment problems at the beginning, and then a subsequent adaptation. There was a suspicious discrepancy between the difficulties reported up until just a moment ago and the contemporary situation. It is likely that those clients wanted to convey their understanding of the importance of the schedule and its positive impact on their behavior, because it was discussed during group therapy sessions. They also demonstrated the tendency to write about their past difficulties, even if the task was to report contemporary experiences. For these reasons they have been included into the group of eight.

**Table 1:** Major common themes of lived time in the novitiate group

Aspect of lived time	Traumatic past (D, E)	Slowed passing (B, E)	Desynchro-nization (A, B, C)	Shortened future horizon (approx.) (E)	Dissociated future (approx.) (E)
Person (gender/age)					
F (m/25)	No	Yes	Yes	1 week	In 2 years/ concrete
J (m/31)	No	Yes	Yes	1 week	Not in time/ abstract
O (m/40)	Yes	No	No	1 week	No
C (m/22)	Yes	Yes	Yes	Less than 1 week	In 1 year/ abstract
Z (m/24)	Yes	Yes	Yes	1 week	Not in time/ abstract
S (m/27)	Yes	Yes	No	Less than 1 week	In 1 year/abstract
B (f/28)	Yes	No	Yes	2 weeks	No
L (f/36)	Yes	Yes	Yes	6 weeks	No
K (m/31)	Yes	Yes	Yes	2 weeks	In 1 year/ abstract
M (f/33)	Yes	No	Yes	Less than 1 week	No

The names of the themes are given by the researcher (letters in parenthesis refer to the method by which it was detected)

### Ad. D

Median scores of the group in ZTPI are given in Table 2. Given the size of the group it was not possible to draw any statistically relevant conclusions. However, it is worth noting that when it comes to the so-called negative past, in contradistinction to all other time orientations, none of the clients had crossed the population median in the direction of the ideal time perspective, the minimum score being 3.2 and maximum being 5.

**Table 2:** Zimbardo Time Perspective Inventory scores of the novitiate group

ZTPI	Negative past	Positive past	Present fatalism	Present hedonism	Future
Novitiate group median	3.7	3.44	2.22	3.8	3.53
Population median according (Zimbardo and Boyd 2008)	3	3.7	2.4	3.4	3.5
Ideal time perspective according (Zimbardo and Boyd 2008)	1.95	4.6	1.5	3.9	4

(*n* = 10; m:f = 7:3; median age = 27.5)

### Ad. E

All clients reacted favorably to the interviews (all have shown an interest in seeing the results of the ZTPI, which were given to them and explained at the beginning of each interview). Following the thematic analysis of the interview notes, based on the intensity and quality of the experiences reported, four major and common thematic phenomena (under the names given by the researcher) have been distinguished in the clients’ lived time; (1) the traumatic past; (2) shortened time horizons; (3) dissociated future; (4) slowed subjective sense of the flow of time . Only a representative sample from the interviews will be given below to illustrate each of the cases. Since these phenomena did not concern all the clients, the exact indication is given in Table 1.

#### Traumatic Past

Eight participants highlighted the unwanted invasion of a negative past affecting their daily present without intentional recollections. The researcher recognized the past as “traumatic” only if it had been reported to be painfully lived through on a daily basis. Regardless of the objectivity of past tragedies, the judgment was based solely on the unpleasant quality of contemporary experiences. Clients reported negative memories, related both to childhood and to experiences with drugs.

C (m/22): “I would like to cut that thing [the past] away, erase it like with a rubber, be like a newborn”. M (f/33): “Everything that I experienced during childhood is bad (…). I have that one memory that haunts me every day… and that opens all the others”. L (f/36): “I freeze on the bad past, it comes by itself… I am trying to suppress it (…). Good things do not come in this way, rather bad things… from my childhood”. Z (m/24): “The past falls upon me every night… it’s like being tormented by the past. I am a martyr type (…). I have no idea how to break away from the past”. Researcher: “Does it happen during the day as well?” Z (m/24): “It happens during small things, at someone’s t-shirt for example (…). I am trying to suppress it with walks, reading books… Every day, I am far away in my thoughts… I am here, but not here”. S (m/27): “It [the past] haunts me often”. R: “How often”? S (m/27): “All the time, in fact…. truly, all the time”. R: “How exactly does it haunt you”? S (m/27): “It will strike once, but hard, and then it disappears”.

Even if it was not a full-fledged theme, it is worth underlining that at least two clients exhibited the tendency to dress their present experiences with past recollections as well as to explain their present as being determined by the past. B (f/28), when asked precise questions about her plans for a job she answered with memories. Being reminded of the question, she started to recall her past again. The story of unfulfilled plans, instead of future plans followed.

#### Shortened Time Horizons

Severely shortened time horizons have been detected: for three clients shorter than a week, for four clients approximately 1 week long, for two other clients about 2 weeks long, with the longest being 6 weeks (see Table 1). Even when explicitly asked to imagine something in the further future, the clients could not come up with any mental images – they had no plans, no hopes, and no desires exceeding their time horizons.

#### Dissociated Future

Despite shortened horizons, the phenomenon of a dissociated future has been observed, and concerned six out of the ten members of the group. The other four had no plans whatsoever, neither abstract nor concrete, exceeding their time horizon (see Table 1). Clients who only briefly mentioned a distant future were not included in this group of six, while those included, speculated extensively about it. What is meant by a dissociated future it is a dream-like plan that is considered realistic while at the same time totally dissociated from the individual’s present – his/her present identity, occupation, available resources, and distance in calendar time. K (m/31), whose plans do not exceed 2 weeks has some ideas about his future after leaving Monar: “It is a form of dream, I believe that something good will come upon me in the future, it motivates me a lot”. F (m/25), who is very low on future time perspective at ZTPI and has an approximately a 1 week long time horizon, has a fixed dream-like future plan, yet it concerns only the distant future. Asked about his plans and expectations for the nearest future, he speaks about becoming a household member. He would also like to speed things up. F (m/25): “I would like to do four sessions in 1 week and start being a household member next week (…). I have a 2 year plan. I am more interested in what will happen afterwards than what happens up to that time”. R: “What is the plan?” F (m/25): “I will finish the therapy, then spend a year abroad, and then open an eatery… Italian cuisine (…). It is the only reason, the only thing why I am here. (…). I do not think that much about the future… the only thing I am thinking about is that restaurant (…). I do not even know how to speak about the future”.

The given example is a perfect case of a dissociated future: it has a concrete form (restaurant with Italian cuisine) and can be placed in calendar time (roughly 2 years in the future). For the other five out of the six clients, who were experiencing the dissociated future, they did not have such a concrete form, but an abstract one. Abstract means that the plans were expressed in a very general way, such as “having a wife and a job” or “living in a great city”, without naming it or indicating the region. This abstract future was either localizable in calendar time (roughly 1 year into the future in three cases) or not even localizable in calendar time (in two cases). The paradox is that all six clients, whose time horizon did not exceed 2 weeks, had “plans” for after the therapy or for its last stages. In other words, in their lived temporality a gap within their future orientation appeared. All six clients fantasized about making a leap over their gap into their distant future.

C (m/22), who is very low on his future in ZTPI, and with a time horizon of less than a week, declares that he has no plans. Yet, when asked about the distant future, he clearly brightens up. Suddenly, he claims to have often planned his distant future, and that the scenarios were positive. C (m/22): “There is this moment every day when I think to myself what I would like to do, what I would do, what I would be occupied with. And at the end of these dreams there comes something like a stone… wooop! And that’s the end… and then again… and again”. R: “Every day, really?” C (m/22): “Every day when I lie in my bed I keep thinking… about what will be after the treatment comes to an end, how to speed it up, how to evade these stages…” R: “How do you feel about it?” C (m/22): “If I was walking on a road without this stone it would be positive (…). I am trying to make up a new system, a new start, so that I do not get addicted again… a new life. R: “How are you going to do this?” C (m/22): “I have no idea. My plan is to jump over these stages… somehow”.

C’s (m/22) plan, like S’s (m/27) and K’s (m/31) is purely abstract, but localizable (after the therapy) while J’s (m/31) and Z’s (m/24) abstract plans are floating outside the calendar: they are not only unable to say anything precise about their content, but also about their temporal locus (in Table 1 as “not in time”) Z (m/24): “I turn myself off every day (…). I walk a lot, and I think about other people, events that are not yet there”.

#### Slowed Passing

Except for two clients of the group of six, all others fantasizing about the distant future felt the concomitant need to accelerate the passing time. As it was already noticed during group discussion (B), their present time was reported to be passing too slowly in comparison with their previous life and was related to the feeling of boredom. Two clients, Z (m/24) and S (m/27), who did not express this need to accelerate explicitly concerning the present, felt it in the context of speaking about the future. Z (m/24) experienced what he himself called “a delusional plan” – to become a household member. S (m/27) also fantasized about being at a much further stage. S (m/27): “I would like to be at the stage of a monarian (…). I would like to be further, at least a caretaker” (see Table 1).

## Discussion

### Results

The main goal of this study was to identify unusual, yet essential lived time experiences of people addicted to multiple drugs when in a sober state. Eight of the clients comprising the study sample experienced difficulties following time in an organized fashion and seven sought to accelerate it. Eight of the clients also experienced an unpleasant domination of their past, while their future horizons appeared significantly shortened and their planning capacity impaired. A distant (dissociated) future was fantasized about in a realistic manner by six of the clients, so that a gap in lived time appeared between a close and a distant future, demonstrating what can be interpreted phenomenologically as a break in continuity of a subjective life history.

Regressive cycles were not observed (probably because the objective time of the study of less than a month was too short). No serious problems with conceptualization of calendar time and no total obstruction of the temporal process have been noticed. Even though all clients had very short temporal horizons, they did not lose the future altogether. Since it remained a dimension of lived time, no total blockage of temporal becoming in the phenomenological sense (von Gebsattel 1954) actually took place.

#### Temporal Orders in Treatment Institutions

Tight scheduling of daily life is a characteristic feature of total institutions (Goffman 1961) and therapeutic communities, where time management constitutes a relevant aspect of treatment with the stated aim of resynchronizing the clients with society (De Leon 2000). Problems with following strict temporal regimes in closed environments and with dealing with free time have been observed (Cope 2003). Addiction has been termed a temporal disorder, resulting in the lack of balance between natural, individual and social time cycles (Jarvinen and Ravn 2015; Ruckenstein 2013).

A Swiss study showed that the slower time passes subjectively, the lower the treatment optimism, with drug-addicted patients having problems following the therapeutic communities temporal order (Klingemann 2001). In the same cultural context, patients upon admission had no grasp of the treatment time horizon and were mostly unable to look forward more than a year (Klingemann and Schibli 2004). Also, Hungarian therapeutic community newcomers scarcely referred to the future and seldom described it in specific terms (Erdos et al. 2009). In Monar-Markot and in Swiss clinics discipline and punctuality were valued, and supportive measures of time organization were considered important for the therapeutic process (Klingemann and Schibli 2004). Conversely, problems with organizing free time, analogical to those experienced by the novitiate group have been interpreted as an obstacle to change in motivational counseling sessions (Sarpavaara 2015).

The temporal aspects of the recovery program at Monar-Markot fulfill the characteristics of resynchronization therapy as described by Fuchs (Fuchs 2001). The rhythms of the daily schedule, leisure activities requiring temporal self-organization, and orientation toward the future enforced through continuous attention to the whole therapeutic program, have rehabilitative value and help to integrate the clients into the lived time of society.

Nevertheless, treatment programs in various institutions differ in terms of their use of time and organizational time orientation, which precludes any direct comparisons (Klingemann and Schibli 2004). Lived time entails a relationship between self and society, with the norms of temporal flexibility for private and institutional time differing between cultures and periods (Jarvinen and Ravn 2015). Also, the pace of life affecting collectively lived time varies between countries and cultures, with Switzerland being the first, Poland the twelfth, and Hungary the nineteenth, according to one comprehensive study (Levine 1997), which makes comparisons difficult.

Monar-Markot clients were not in a state of recovery, which is temporarily characterized by a clear difference between the past and the present, as well as by the opening of future horizons (Banonis 1989; Hanemaayer 2009). For them, the past was conspicuously present – a phenomenon also found in the Hungarian case (Erdos et al. 2009). This was not a positive situation, all the more so since within the tradition of phenomenology it has been agreed that inside the threefold time extension a future-orientation is the most important (von Gebsattel 1954; Heidegger 1962; Minkowski 1970; Mooij 2005; Moskalewicz 2014).

#### Dissociated Future and Ecstasy

By far the most interesting phenomenon detected was the dissociated future. However, in order to confirm the existence of this phenomenon, further research is needed. A dissociated future can be interpreted as a result of an addicted person trying to break away from the painful past and the empty present (von Gebsattel 1954). Since people suffering from addiction live inauthentically and try to escape from themselves (Hirschman 1992), a dissociated future might be an example of an attempt to run away mentally from such an inauthentic existence. The weight of the past along with a shortened time horizon, rendering the lived present difficult to bear, may lead to “utopian phantasies”. These fantasies resemble what Rollo May called an “opiate form of hope”, i.e. one that differs from normal hope in the way that it breaks the bond between the present and the future (May 1953). Therefore, we may speculate, a dissociated future has a compensatory function, though it might well be a normal experience of people who are in a difficult situation and simply dream extensively about a better life.

In addition, if we look at the experience of drug-induced ecstasy from the temporal perspective, we will find that it consists of immersion in a purely theoretical potentiality. It has been argued in detail that the paradox of ecstasy is that one experiences many unrealizable possibilities (Zutt 1963b). In this sense, a dissociated future might be rationalized as a leftover from previous ecstasies, a temporal experience phenomenon that became a part of the sober self. Nevertheless, it can only be speculated whether this phenomenon subsequently developed into drug use or was a prior selective factor in the original development of the addiction.

#### Conclusion and its Limits

It may be concluded that the lived time of addiction therapy newcomers was slowed down, with the past seriously outweighing the future, so that their experience of time overall might be seen as having clear depressive traits. In addition to already known experiences of desynchronization and shortened future horizons, the phenomenon of a distant, dissociated future, being fantasized about in a realistic manner, has been detected, breaking the continuity of the lived time to an extent.

Nevertheless, since the group was small and the clients addicted to multiple drugs, no inferences should be drawn regarding the prevalence of observed time disturbances beyond the given sample. The contextual validity of the results must also be kept in mind, including certain limiting factors, primarily the lack of ethnic and cultural diversity in the group. Particularly, it is not known whether the phenomenon of the dissociated future in the aforementioned sense can be found in other groups of addicted individuals. In addition, even if the therapeutic community is supposed to and was assumed to represent the everyday life-world, it is clearly an artificial and not a real, life-world environment, with stricter rules of conduct than the social reality outside, which could have affected the findings.

A repetition of the study was planned using the same methods with the same group after a year (at graduation) in order to check if the treatment changed the quality of the clients’ lived time (Banonis 1989; Davies and Filippopoulos 2015). However, the follow up was impossible since only one member of the novitiate group made it to the end. It was O (m/40) – the oldest of the group and the only one who, despite his traumatic past, experienced neither a dissociated future, the need to accelerate the passing time nor temporal adjustment difficulties.
